# Genomic screening of testicular germ cell tumors from monozygotic twins

**DOI:** 10.1186/s13023-014-0181-x

**Published:** 2014-11-26

**Authors:** Sara Martoreli Silveira, Isabela Werneck da Cunha, Fabio Albuquerque Marchi, Ariane Fidelis Busso, Ademar Lopes, Silvia Regina Rogatto

**Affiliations:** Neogene Laboratory, CIPE, A. C. Camargo Cancer Center, São Paulo, Brazil; Department of Pathology, A.C. Camargo Cancer Center, São Paulo, Brazil; Institute of Mathematics and Statistics, Inter-Institutional Program on Bioinformatics, USP, São Paulo, Brazil; Nucleus of Sarcoma, Department of Pelvic Surgery, A.C. Camargo Cancer Center, São Paulo, Brazil; Department of Urology, Faculty of Medicine, UNESP, Botucatu, São Paulo, Brazil

**Keywords:** Testicular germ cell tumors, Seminomas, Embryonal carcinoma, Array-based comparative genomic hybridization, Molecular markers, Genomic imbalances, Copy number variations

## Abstract

**Background:**

Testicular germ cell tumors (TGCTs) account for 1-2% of all tumors in young and middle aged men. A 75-fold increase in TCGT development has been reported for monozygotic (MZ) twins. Therefore, the occurrence of simultaneous tumors in MZ twins emphasizes the importance of genetic factors that influence the risk of developing these tumors. Genomic screening was performed for one family containing MZ twins with testicular germ cell tumors, in order to define alterations associated with risk of tumor development.

**Methods:**

Copy number alterations were evaluated using array-CGH (4x44K, Agilent Technologies) in one seminoma and one embryonal carcinoma (EC) from MZ twins. In addition, genomic alterations from the tumors and peripheral blood cells of the twins were compared to the parental genomes via their peripheral blood cells.

**Results:**

Embryonal carcinoma (Twin-1 t) presented a lower frequency of genomic alterations compared to the seminoma (Twin-2 t). One minimal common region of loss was observed in 9p13.1-p12 in the comparison between DNA from blood samples for Twin-1 and Twin-2. In this region is mapped the *CNTNAP3* gene which was confirmed as involved in losses by qPCR. Comparative analysis of novel CNVs between the Twin-1 t and Twin-2 t showed five minimal common regions involving gain at chromosomes 12 (12p12.3-p11.1 and 12p13.33-p12.3), while losses were observed at 10p15.3-p15.2, 13q21.1-q21.2 and 15q11.1-q11.2. In addition, one exclusive rare copy number alteration was detected in Twin-1 t and Twin-2 t, and 19 novel alterations were identified in the Twin-2 t.

**Conclusion:**

Distinct genomic profiles for MZ twins with phenotypically different TGCT were described. Of particular interest, 12p gains were detected exclusively in tumor samples. In peripheral blood samples, loss of 9p13.1-p12 was the unique novel CNV shared by the twins, confirming the involvement of *CNTNAP3* gene in TGCTs development. Although similar CNV profiles were shared by both the peripheral blood and tumor samples of the twins, tumor-specific CNV loci were identified for seminoma and non-seminomatous tumors. These findings suggest the presence of *de novo* germline structural alterations and TGCT predisposition.

**Electronic supplementary material:**

The online version of this article (doi:10.1186/s13023-014-0181-x) contains supplementary material, which is available to authorized users.

## Background

Testicular tumors are relatively uncommon, accounting for 1-2% of all tumors in males. Testicular germ cell tumors (TGCTs) represent 95% of testicular cancers and are histologically classified as seminoma or non-seminoma [1]. Seminoma presents a homogenous histological pattern that resembles primordial germ cells (PGCs) [2]. Non-seminomatous tumors are classified as embryonic (teratoma) and extra-embryonic (yolk sac and choriocarcinoma) according to their pattern of cell differentiation, or as embryonal cell carcinomas when originating from undifferentiated cells [3,4]. It is important to highlight that non-seminomatous mixed germ cell tumors display a more aggressive clinical behavior, frequently developing local recurrence and distant metastasis [5].

The presence of these tumors in monozygotic (MZ) twins is extremely rare and therefore, it is difficult to assess the impact and risk associated with inheritance. Monozygotic and dizygotic twins present a 75 and 35-fold increased risk of developing TCGT, respectively [6]. In a meta-analysis of testicular cancer in twins, two of seven studies demonstrated a significantly increased risk of testicular cancer among twins [7]. Therefore, a simultaneous occurrence of tumors in MZ twins stresses the importance of a genetic factor, which influences their risk of development.

The etiology of testicular cancer is not completely understood, however, a number of risk factors have been described in the literature, including cryptorchidism, a family history of testicular cancer, testicular atrophy, testicular microlithiasis, infertility, HIV infection and imbalance of estrogen and androgen levels in twin pregnancies [8-11]. In addition, rare inherited diseases, such as Down’s syndrome, Klinefelter’s syndrome, and XY dysgenesis, are associated with an increased risk for testicular cancer [12]. Gains at Xq27, *XRCC1* polymorphisms and deletions of the Y chromosome have been described as associated with susceptibility to testicular germ cell tumor [13-17]. Recent genome-wide association studies (GWAS) have identified 29 SNPs mapped at 18 TGCT predisposition loci [18-22]. Several genes have been described as associated with TGCT susceptibility including *TERT, SPRY4, BAK1, DMRT1, ATF7IP* and *KITLG*.

In the present study, a genomic screening approach was performed for a pair of 26-year old male MZ twins with embryonal carcinoma and testicular seminoma. Copy number variations (CNV) analyses were presented and discussed with reference to the parental genome, which allowed for the assessment of genomic alterations and respective harbored genes involved in the development of the testicular germ cell tumors.

## Methods

### Case description

A pair of male MZ twins with a diagnosis of TGCTs was admitted to the Sarcoma’s Nucleus of the A. C. Camargo Cancer Center, São Paulo, Brazil. Array-CGH analysis was successfully performed for the primary tumors (embryonal carcinoma and seminoma) and the peripheral blood samples from the twins and their parents. Neither patient had received chemotherapy or radiotherapy prior to sample collection. Furthermore, neither twin presented risk factors associated with TCGT as described in the literature (cryptorchidism, familial testicular cancer history, testicular atrophy, testicular microlithiasis, infertility, HIV infection and alterations of estrogen and androgen levels during pregnancy). All family members were advised of the procedures and provided written informed consent. The Human Research Ethics Committee of the Institution approved the study (Protocol EC 21/2014).

**Figure 1:**
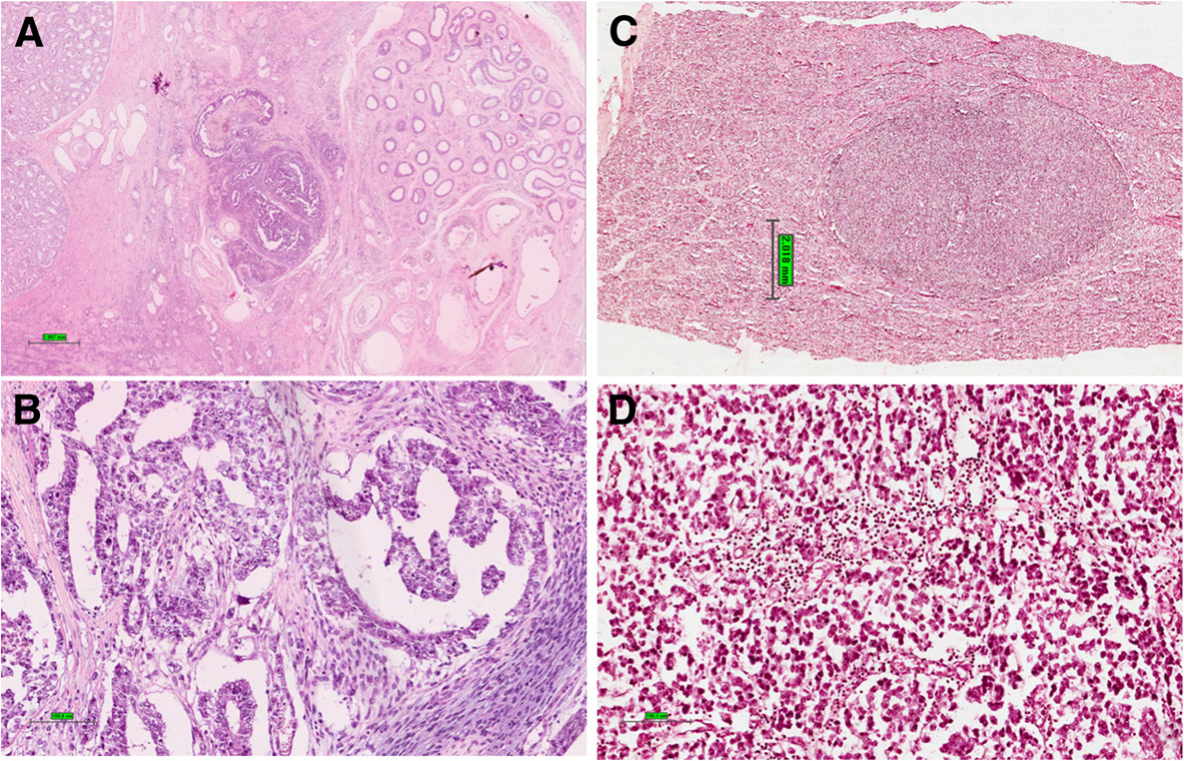
**Identical twins diagnosed with different subtypes of testicular germ cell tumors. A)** Embryonal carcinoma. **B)** Embryonal carcinoma in detail, showing a papillary growth pattern. **C)** Seminoma nodule within the testis **D)** Seminoma in detail, showing large round cells with lymphocytes.

Twin-1 was submitted to a right orchiectomy after presenting with a palpable tumor in the right testicle at 21 years of age. Histopathological analysis revealed a mixed germ cell tumor (pT2NxMx) containing both embryonal carcinoma (95%) and mature teratoma (5%) components (Figure 1A-B). Immunohistochemistry for the embryonal carcinoma was positive for CD30 and negative for PLAP, HCG and alpha- fetoprotein. Despite the presence of lymphatic and venous invasion, the adjacent testicular anatomy (tunica albuginea and vaginalis) was not compromised and no invasion of the spermatic cord was observed. The surgical margins were negative for the presence of tumor cells. Due to the presence of pulmonary metastases, the patient underwent a three-day regimen of adjuvant chemotherapy with bleomycin, etoposide and cisplatin (BEP), with a complete response. Three years after the initial diagnosis, a new nodule was detected in the left testicle. Histopathological evaluation revealed a mature teratoma, clinical stage IA. Subsequently, the patient had a left orchiectomy and after 6 years of follow-up remains disease-free.

Twin-2 was submitted to a right orchiectomy at 22 years of age, after presenting with a palpable tumor in the right testicle. Histopathological analyses revealed a pure classical seminoma (pT1NxMx) (Figure 1C-D) with no vascular or lymphatic invasion. The adjacent testicular anatomy (rete testis, epididymis, tunica albuginea, tunica vaginalis and spermatic cord) was not compromised. It was pathologically staged as 1A, indicated a good prognosis; therefore no adjuvant treatment was required. Fifteen months later, testicular ultrasound detected a new solid nodule in the left testicle measuring 0.5 cm, associated with microlithiasis, diagnosed as a classic seminoma, without any other germ cell component. The patient underwent wedge resection of the tumor nodule and adjuvant radiotherapy (25.5 Gy) was administered to the remaining testis. After four years of follow-up, the patient remains disease-free.

### Genome screening using array-based comparative genomic hybridization (array- CGH)

Formalin fixed paraffin-embedded tumors, one embryonal carcinoma (Twin-1 t) and one seminoma (Twin-2 t), were macro-dissected and genomic DNA extracted using a standard phenol/chloroform method. The same protocol was applied for DNA extraction from peripheral blood from the twins and their parents. The Peripheral blood samples, tumors and reference genomic DNA (Promega, G1471, male) were differentially labeled using a Genomic DNA Enzymatic Labeling Kit (Agilent Technologies, Santa Clara, CA, USA). Hybridization was performed using Agilent Human CGH 44 K Oligo Microarrays according to the manufacturer’s instructions. The array images were acquired using a DNA microarray scanner with Surescan High- Resolution Technology (Agilent Technologies, Santa Clara, CA) and Scan Control (version 8.1, Agilent Technologies) software. The data were extracted and normalized using the Feature Extraction software (version 10.1.1.1, Agilent Technologies). Statistical analysis was performed using the Nexus Copy Number software (version 7.5 – Hg19, Biodiscovery Inc., El Segundo, CA, USA). The Fast Adaptive States Segmentation Technique 2 (FASST2) algorithm and Significance Testing for Aberrant Copy number (STAC) statistical methods were used to identify non-random genomic copy-number alterations [23]. According to these algorithms, DNA copy number alteration was defined as follows: exceeding the significance threshold of 1×10^−5^; containing at least three consecutive altered probes per segment; and specified 1000 kb as being the maximum spacing between adjacent probes. These parameters were used to define the values for copy number gains (log2 ≥ 0.2), high copy number gains (log2 ≥ 0.6), losses (log2 ≤ −0.2) and homozygous losses (log2 ≤ −1.0).

### Copy number variation analyses

The altered genomic regions observed were initially compared with the CNV described in the Database of Genomic Variants (DGV) (http://dgvbeta.tcag.ca/dgv/app/home?ref=GRCh37/hg19/, November 2013) and then with a CNV reference obtained from a database of the Brazilian population [24]. The CNVs were subsequently classified according to their genomic size and polymorphic frequency as follows: common (regions larger than 100 kb and often present in DGV frequencies greater than or equal to 1%); rare (regions larger than 100 kb and often present in DGV frequencies less than or equal to 1%); and novel (regions larger than 100 kb and not yet described in the DGV). This study focused only on rare or novel CNVs. Despite the majority of the CNV described in this study having a partial intersection with the common regions identified in the DGV database, the total CNV length had not yet been described in either the DGV or the Brazilian database. In addition, only genes mapped in these regions not covered by common CNVs were considered.

### Quantitative real-time PCR (qPCR) analysis

The genomic DNA sequences of candidate region were obtained from the Ensembl Genome Browser website (GRCh37/hg19 Human Reference Assembly; February 2009). The reactions were performed in triplicate and followed PCR cycling conditions as previously described by [25]. Briefly, primer pair of *CNTNAP3* gene (covering the same probes represented in the 4x44K Agilent plataform) was designed to amplify the altered regions detected by using array-CGH (*Forward: TCCAGACAGATGAGCAAAACATA; Reverse: AGAAAGAAAGACAACTCTCTGCT*). Delta-delta Ct model was used to calculated the relative copy numbers [26] based on the *CNTNAP3/HPRT* (reference gene) ratio. This value was defined as a loss when the ratio was <0.68 and as a gain when the ratio was >1.47.

## Results and discussion

There is a strong hereditary component in testicular germ cell tumors as demonstrated by twin studies [6,7,27,28]. Over the past 10 years, pertinent advances have been reported in MZ twin CNV studies, revealing an association with disease susceptibility, including neurological disorders (amyotrophic lateral sclerosis, Parkinson disease and Lewy body dementia) and psychiatric disorders (autism, bipolar disorder and schizophrenia) [29-32]. The present study is thought to be the first report demonstrating the novel and rare CNV profiles of a pair of MZ twins with different testicular germ cell tumor phenotypes. The study design was used for both the peripheral blood samples from the twins and their parents as well as the tumors (seminoma and embryonal carcinoma) from twins.

### Comparison between constitutional CNVs from parents and siblings

The array-CGH profiles obtained from the genomic DNA of the peripheral blood from the father (Parent-1) and the mother (Parent-2) were compared to their sons (Twin-1b and Twin-2b). Parents and siblings shared similar patterns in terms of common CNVs. Rare or novel CNVs were not detected during these comparisons, most likely due to the low resolution of the platform. The peripheral blood samples from the MZ twins presented a discordant genotype profile, suggesting the contribution of post- zygotic events. The differences in genetic profiles of MZ twins has been previously characterized according to alterations involving the number or morphology of chromosomes, chromosomal mosaicism, single nucleotide polymorphisms and epigenetic modifications [33]. In this study, a panel of potentially pathogenic and novel CNVs was identified by array-CGH in each twin (Tables 1 and 2).

**Table 1:** **Significant copy number variation (CNVs) regions detected by array-CGH**

**Cytoband** ^**#**^	**Samples**		**Start**	**Stop**	**Length (pb)**	**Event**	**N°**	**Gene ID**
	**Blood**	**Tumor**						
9p12-p13.1	Twin-1b		38.944.256	40.498.819	1554563	Loss	7	*CNTNAP3, SPATA31A1, SPATA31A2, ZNF658B, LOC653501, DQ586551* and *CNTNAP3B*
	Twin-2b							
10p15.3-p15.2		Twin-1 t	1,056,859	3,168,906	2112047	Loss	18	*ADARB2, ADARB2-AS1, AK097474, AK127347, AX748285, IDI1, IDI2, IDI2-AS1, PFKP*, WDR37*
		Twin-2 t						
12p12.3-p11.1		Twin-1 t	15.161.204	34.278.525	19117321	Gain	126	PLCZ1, CAPZA3, CASC1, KRAS, ASUN, PPFIBP1, DDX11, MANSC4, KLHL42, PTHLH*
		Twin-2 t						
12p13.33- p12.3		Twin-1 t	0	14.900.955	14900955	Gain	351	ACRBP, AKAP3, CCND2, DDX47, LRP6, NANOG, YBX3, CACNA1C, FGF6, FKBP4*
		Twin-2 t						
13q21.1-q21.2		Twin-1 t	58.321.428	60.493.818	2172390	Loss	6	*AK097816, BC032915, CR618830, DIAPH3, TDRD3* and *DIAP3*
		Twin-2 t						
15q11.1-q11.2		Twin-1 t	20.335.887	23.044.681	2708794	Loss	140	SNORD116I, SNORD115, SNORD107, SNORD64, MAGEL2, NDN, SNURF, PAR-SN, PAR5, SNORD108*
		Twin-2 t						
Xq27.3	Twin-2b	Twin-2 t	144.344.622	145.204.327	859.706	Gain	9	*SLITRK2, TMEM257, MIR892C, MIR890, MIR888, MIR892A, MIR892B, MIR891B* and *MIR891A*
Xq28	Twin-1b	Twin-1 t	150.522.209	153.914.671	3.392.463	Gain	7	*MAGEA2, MAGEA2B, FATE1, LAGE3, CTAG1B, CTAG1A and CTAG2*

Legend: #: It was found only novel CNVs in the blood and tumor samples from monozygotic twins (DGV and Brazilian Database). *More than 10 genes were mapped in this representative chromosome region. The complete gene list is deposited in Gene Expression Omnibus (GEO) database (www.ncbi.nlm.nih.gov/geo/). Accession number: GSE62779.

**Table 2:** **Specific rare and novel CNVs detected in embryonal carcinoma (Twin-1t) and seminoma (Twin-2t)**

**Tumor Sample**	**Cytoband**	**Start (pb)**	**Stop (pb)**	**Size (pb)**	**Event**	**CNV Classification** ^**a**^	**Genes** ^**b**^
**Twin-1 t**	16p11.2-p11.1	34.059.589	34.847.384	787796	Gain	R	NI
**Twin-2 t**	1p31.1	77.762.182	78.226.565	464384	Gain	N	NI
	1p22.1	93.075.850	93.586.348	510499	Gain	N	NI
	2p23.3	27.431.951	27.496.511	64561	Gain	N	*EIF2B4*
	2q33.1	201.865.021	202.014.428	149408	Gain	N	NI
	3q13.13	110.529.591	110.874.657	345067	Loss	N	NI
	3q13.33	120.713.729	120.813.244	99516	Gain	N	NI
	6p21.1	42.257.603	42.545.298	287696	Gain	N	*GUCA1A,GUCA1B, MRPS10* and *TRERF1*
	8p11.23	39.003.502	39.157.973	154472	Loss	N	*ADAM9* and *ADAM32*
	10q24.1-q24.2	99.386.286	99.516.551	130266	Gain	N	*PI4K2A, AVPI1, MARVELD1, ZFYVE27* and *SFRP5*
	12q13.3-q14.1	56.296.387	56.308.803	12417	Loss	N	NI
	12q24.31	119.381.319	119.652.493	271175	Gain	N	NI
	14q11.2	20.550.697	20.622.417	71721	Loss	N	NI
	14q23.1	58.887.110	59.149.379	262270	Loss	N	NI
	15q25.3-q26.1	86.730.625	86.986.408	255784	Gain	N	*MRPL46, MRPS11, DET1, MIR1179, MIR7-2, MIR3529, AEN* and *ISG20*
	17p11.2	19.968.313	20.108.133	139821	Loss	N	NI
	17p13.1	7.423.749	7.459.204	35456	Gain	N	NI
	19q13.43	62.712.083	62.882.866	170784	Gain	N	*ZNF773, ZNF549, ZNF550, ZNF416, ZIK1, ZNF530, ZNF134, ZNF211* and *ZSCAN4*
	20q11.21	29.352.138	29.447.677	95540	Gain	N	*DEFB116, DEFB118* and *DEFB119*
	Xp22.12-p22.11	20.043.034	21.803.101	1760068	Gain	R	*SCARNA9L, SMPX, YY2* and *MBTPS2*

Legend: CNV – copy number variation; Twin-1 t – embryonal carcinoma; Twin-2 t – seminoma. Rare – R; Novel – N; No identified – NI. a - CNV classification based on the Database of Genomic Variants and in the Brazilian population reference database. b- Genes mapped in regions described as rare or novel CNV.

### Comparison between the genomic profiles of the blood and tumor from Twin-1

The comparison between the genomic alterations found in the peripheral blood and tumor samples from Twin-1 revealed the presence of minimal regions of gain at Xq28. The Xq28 locus harbored seven genes (*MAGEA2, MAGEA2B, FATE1*, *LAGE3, CTAG1B, CTAG1A* and *CTAG2*), three of which are responsible for encoding the cancer-testis antigens *CTAG1B, CTAG1A* and *CTAG2*. The cancer-testis antigens (CTA) are highly expressed in normal germ line cells as well as in many cancers, where they are associated with advanced disease and a poor prognosis [34,35]. Several studies have demonstrated that CTA production in tumor cells occurs due to reactivation of the gametogenic expression program as result of genomic instability [35-37]. In the present study, gains of Xq28 CTA-cluster in embryonal carcinoma and blood samples obtained from Twin-1 were identified. This cluster was not observed for the seminomatous from Twin-2.

### Comparison between the genomic profile in blood and tumor from Twin-2

The peripheral blood and tumor samples from Twin-2 shared novel CNV involving gains at Xq27.3. This CNV was not detected in embryonal carcinoma from Twin-1. Nine genes were mapped on Xq27.3, including six miRNAs (*miR890, miR888, miR891A, miR891B, miR892A* and *miR892B*). *miR890* and *miR888* have previously been described as body fluid-specific, only detected in semen and epididymal tissue [38]. However, no association has been described for TGCT. Although these findings are interesting, further studies are required in order to confirm that the alterations involving these miRNAs are associated with risk of developing TGCT.

### Comparison between the peripheral blood samples from Twin-1 and Twin-2

**Figure 2:**
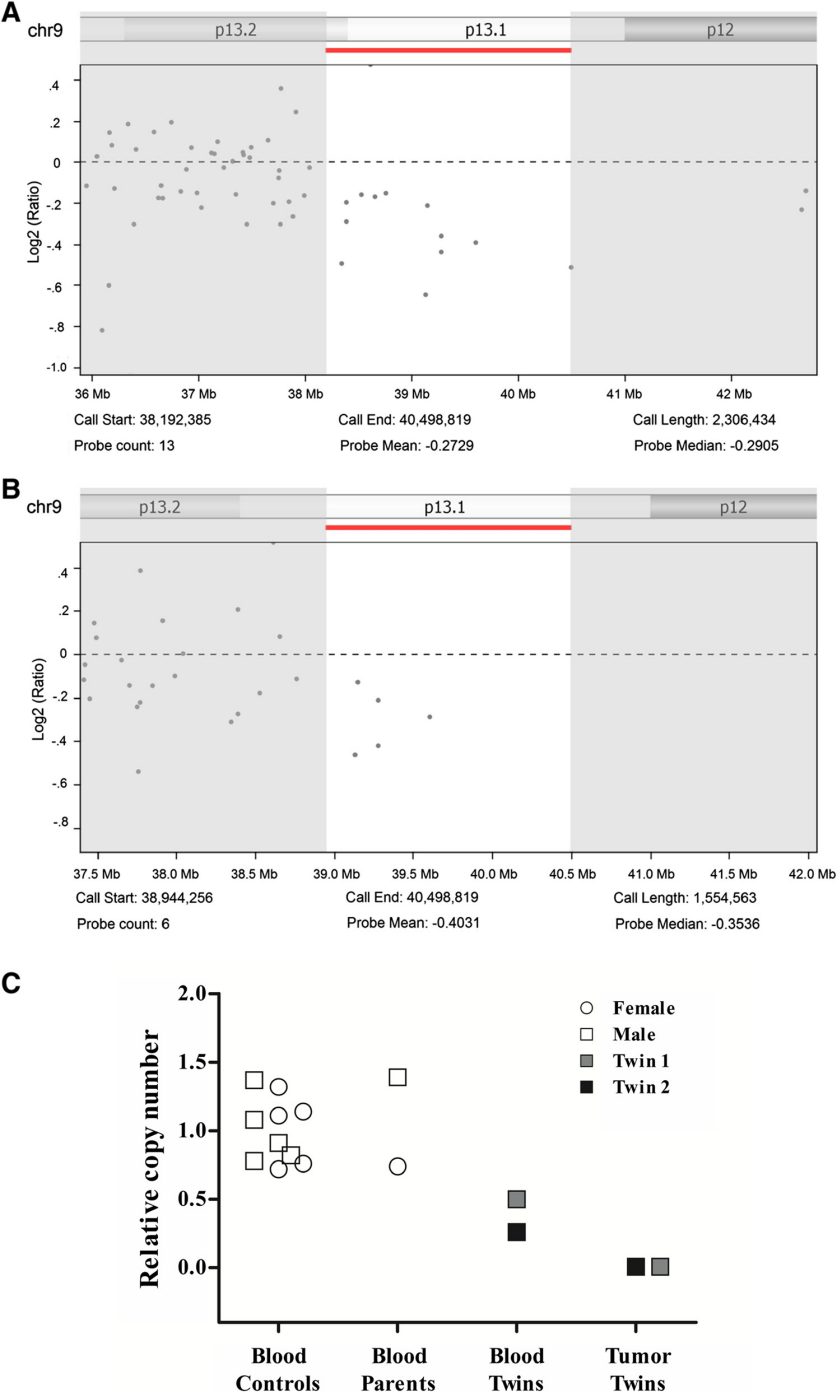
**Array-CGH profile showing loss at 9p13.1-p12 was confirmed by qPCR for** ***CNTNAP3*** **gene. (A**-**B)** 9p13.1-p12 loss was the unique novel CNV shared by the Twin-1 **(A)** and Twin-2 **(B)**. Scatter plots with x-axis coordinate representing the probes positions along the genome. The low bars (red) indicate losses of genomic regions. The images were adapted from the Nexus 7.5 software. **(C)** Relative quantification of *CNTNAP3* copy number alterations evaluated by qPCR. Normal DNA copy number (two copies) was found in the controls and parental blood. Losses were found in peripheral blood and tumor cells from twins. Image obtained from Graphpad Prism 5 (Graphpad Software Inc., La Jolla, CA).

Although 9p12-p13.1 losses were covered by common CNVs, the region remained included for analysis due to its presence in blood of Twin-1 and Twin-2 (Figure 2). In addition, this region harbors potential candidate genes that have been previously described in TGCT. Seven genes were mapped in this region, including two members of the *SPATA31* (AEP1) subfamily (*SPATA31A1* and *SPATA31A2*) and *CNTNAP3*. The *SPATA31* testis-specific gene codified an integral component of membrane that has been associated to spermatogenesis and the cell differentiation process, possibly playing a role in acrosome formation [39,40]. *CNTNAP3* gene encodes a protein associated to cell-recognition process within the nervous system [41]. Although no significant by the criteria used in our study, loss of *CNTNAP3* was detected by array-CGH in both tumor samples from twins (two consecutive probes). Quantitative real-time PCR (qPCR) was performed to confirm the loss at 9p13.1-p12 involving the *CNTNAP3* gene. Relative DNA copy number loss was found in both, blood and tumor (homozygous deletion) from twins (Figure 2). The controls and parental blood samples showed relative DNA copy number within the reference interval. The qPCR analysis confirmed the array-CGH results as well as the homozygous deletions found in tumor samples. Overall, these data suggest that the genes mapped on 9p12-13.1 have a strong potential to be involved in testicular tumor development.

### Panel of novel CNVs shared by seminoma and embryonial carcinoma

**Figure 3:**
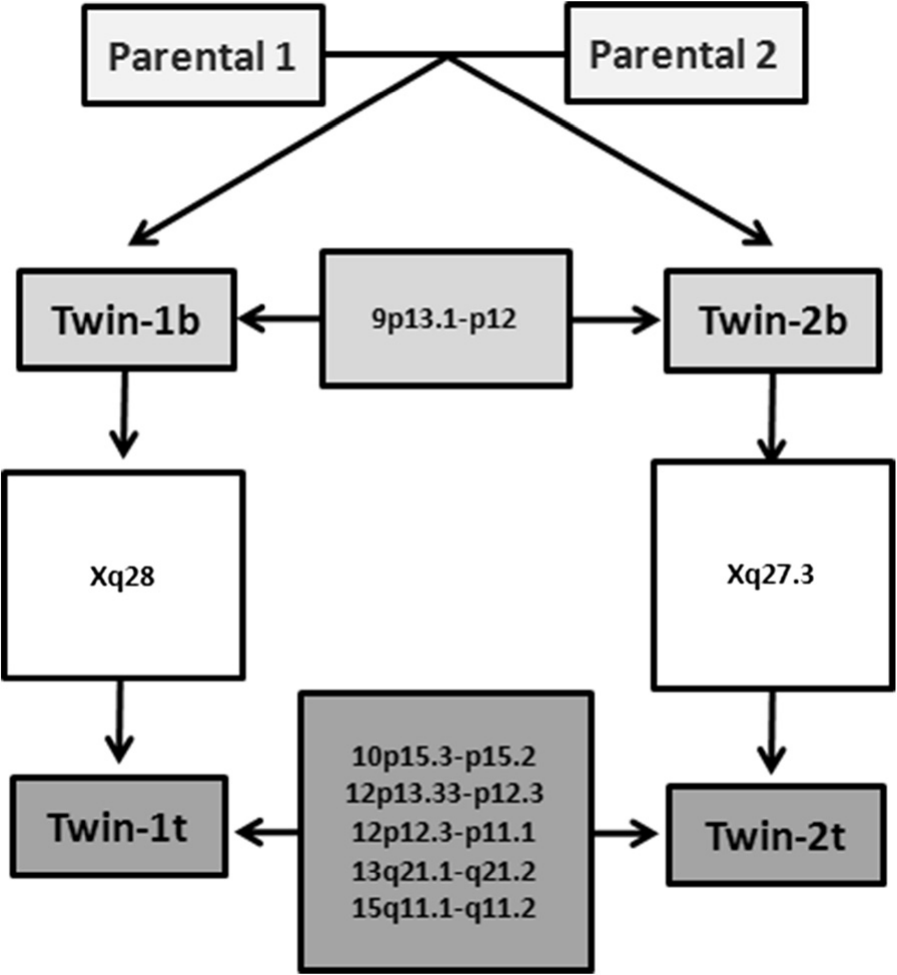
**Schematic representation of the genomic alterations detected in peripheral blood cells (b) and tumors (t) from the parents and twins.** In peripheral blood, loss of 9p13.1-p12 was the unique novel CNV shared by the twins. Two specific CNVs mapped in chromosome X were shared by both tumor and blood samples of each sibling, including Xq28 for Twin-1 and Xq27.3 for Twin-2. The panel of novel CNVs shared by embryonal carcinoma (Twin-1 t) and the seminoma (Twin-2 t), including gains at 12p (12p12.3-p11.1 and 12p13.33-p12.3), as well as losses at 10p15.3-p15.2, 13q21.1-q21.2 and 15q11.1-q11.2. None of these novel CNVs were shared with the parental genotypes.

The presence of rare or novel CNVs detected in seminoma and embryonal carcinoma suggests a common mechanism that contributes to tumorigenesis. Comparative analysis of novel CNVs from the embryonal carcinoma and seminoma revealed five minimal common regions, involving gain at chromosome 12 (12p12.3-p11.1 and 12p13.33-p12.3), while losses were observed at 10p15.3-p15.2, 13q21.1-q21.2 and 15q11.1-q11.2 (Figure 3).

DNA copy number alterations involving 12p have been described in both seminoma and non-seminoma. Amplification of 12p, as observed in the tumor samples from Twin-1 and Twin2 (Additional file 1), was first described by Atkin et al. [42], and subsequently in other studies [43-46]. Gains of 12p have been detected in primordial cells responsible for determining intratubular germ cell neoplasia of the unclassified type, suggesting that possession of this alteration contributes to an invasive phenotype [47-49]. Several candidate target genes have been mapped to this region, including *KRAS* and *CCND2,* which have been associated with the malignant transformation process via the activation of pathways involved in cell proliferation [48,50], or in the maintenance of germ cell pluripotency, such as *DPPA3* and *NANOG* [46,51]. Furthermore, we identified several spermatogenesis-associated genes mapped in 12p12.3-p11.1 and 12p13.33-p12.3 including *ACRBP, AKAP3, DDX47, PLCZ1, CAPZA3, ASUN* and *DDX11.*

Eighteen genes mapped at deleted region 10p15.3-p15.2 including *PFKP, ADARB2* and *WDR37*. In agreement with our findings, Hofer et al. [52] reported PFKP down-expression among 55 differently expressed genes in seminomas and embryonal carcinomas. A high incidence of 13q deletion has been reported in primary TGCTs [43,44,53] and in non-seminomatous germ cell tumours with acquired treatment resistance [53]. In our study, it was detected losses of 13q21.1-q21.2, which harbors seven genes, including *DIAPH3, TDRD3* and *DIAP3*. Loss of 15q11.1-q11.2 was also observed for both tumors, and involved the *SNORD* small nucleolar RNA (snoRNA) cluster. The *SNORD116I* and *SNORD115* genes belong to the C/D box family of snoRNAs, and present a number of important regulatory RNA editing functions. Further studies are necessary to clarify the involvement of these small RNAs on the development of TGCT.

### Panel of the rare and novel tumor-specific CNVs

The comparative analysis of seminoma and embryonal carcinoma obtained from MZ twins revealed a panel of two rare and 19 novel tumor-specific CNVs (Table 2). To our knowledge, four studies evaluated copy number variations in TGCT [54-57]. Using array-CGH, Edsgärd et al. [54] analyzed the profile of pathologic CNVs in families with germ cell tumors, including TGCT. However, only common variants were identified, including heterozygous deletion involving *RLN1* (9p24.1). Subsequently the authors had evaluated common and rare CNVs in a TGCC case–control cohort, but no single locus was found to be associated with these tumors. Instead, the authors described one potential deletion of *PTPN1* gene (mapped at 20q13) as rare CNVs [55]. Stadler et al. [56] described three de novo CNV events involving amplification of 7q11.22 and 12q24.1, and loss of 6p21.2 in 3/43 familial testicular germ cell tumor trios evaluated. In a cohort of 212 cases of TGCT and 437 controls, Dalgaard et al. [57] reported a weak association for rare CNV in relation to cell migration in TGCT. These altered-locus or genes were not detected in our analysis. In agreement to the previous studies, our data suggest an association between several *de novo* germline structural aberrations and TGCT predisposition rather than one rare event influencing the phenotype.

## Conclusion

This study constitutes the first report describing rare and novel CNVs of TGCT in monozygotic twins with divergent genomic profiles. Several CNV regions that harbor crucial regulatory genes were associated with TGCT pathogenesis. Loss of *CNTNAP3* mapped at 9p13.1-p12 was confirmed as involved in TGCT development. Of particular importance, a pathognomonic gain of 12p was confirmed exclusively in tumor samples, which was not observed in the peripheral blood of the twins or their parents. Although similar CNV profiles were shared by for both the peripheral blood and tumor samples of the twins, tumor-specific CNV loci were identified for seminoma and non- seminomatous tumors, which suggest a CNV origin for *de novo* events.

## Availability of supporting data

The data set supporting the results of this article is available in the Gene Expression Omnibus (GEO) repository (www.ncbi.nlm.nih.gov/geo/). Accession number: GSE62779.

## Additional file

Additional file 1: Figure S1. Hybridization profile for 12p amplification identified in tumor samples of twins. In embryonal carcinoma (A-B) and the seminoma (C), gains at 12p were important novel CNV mapped at 12p amplicon. The 12p12.3-p11.1 (A) and 12p13.33-p12.3 (B) were detected in Twin-1 and 12p13.33-p11.1 (C) amplicon was detected in Twin-2. Scatter plots with x-axis coordinate representing the probes positions along the genome. The top bars (blue) indicate gains of genomic regions, whereas the lower bars (red) indicate losses of genomic regions. The images were adapted from the Nexus 7.5 software.

## Abbreviations

TGCT: Testicular germ cell tumors
CNV: Copy number variation
MZ: Monozygotic twins
EC: Embryonal carcinoma
