# Supplementary material for: Genomic screening of testicular germ cell tumors from monozygotic twins
Source: Orphanet J Rare Dis. 2014 Nov 26;9:181. doi: 10.1186/s13023-014-0181-x (PMC4254261; doi:10.1186/s13023-014-0181-x)
Supplement: Additional file 1: Figure S1. — Hybridization profile for 12p amplification identified in tumor samples of twins. In embryonal carcinoma (A-B) and the seminoma (C), gains at 12p were important novel CNV mapped at 12p amplicon. The 12p12.3-p11.1 (A) and 12p13.33-p12.3 (B) were detected in Twin-1 and 12p13.33-p11.1 (C) amplicon was detected in Twin-2. Scatter plots with x-axis coordinate representing the probes positions along the genome. The top bars (blue) indicate gains of genomic regions, whereas the lower bars (red) indicate losses of genomic regions. The images were adapted from the Nexus 7.5 software. [file 13023_2014_181_MOESM1_ESM.docx]

**
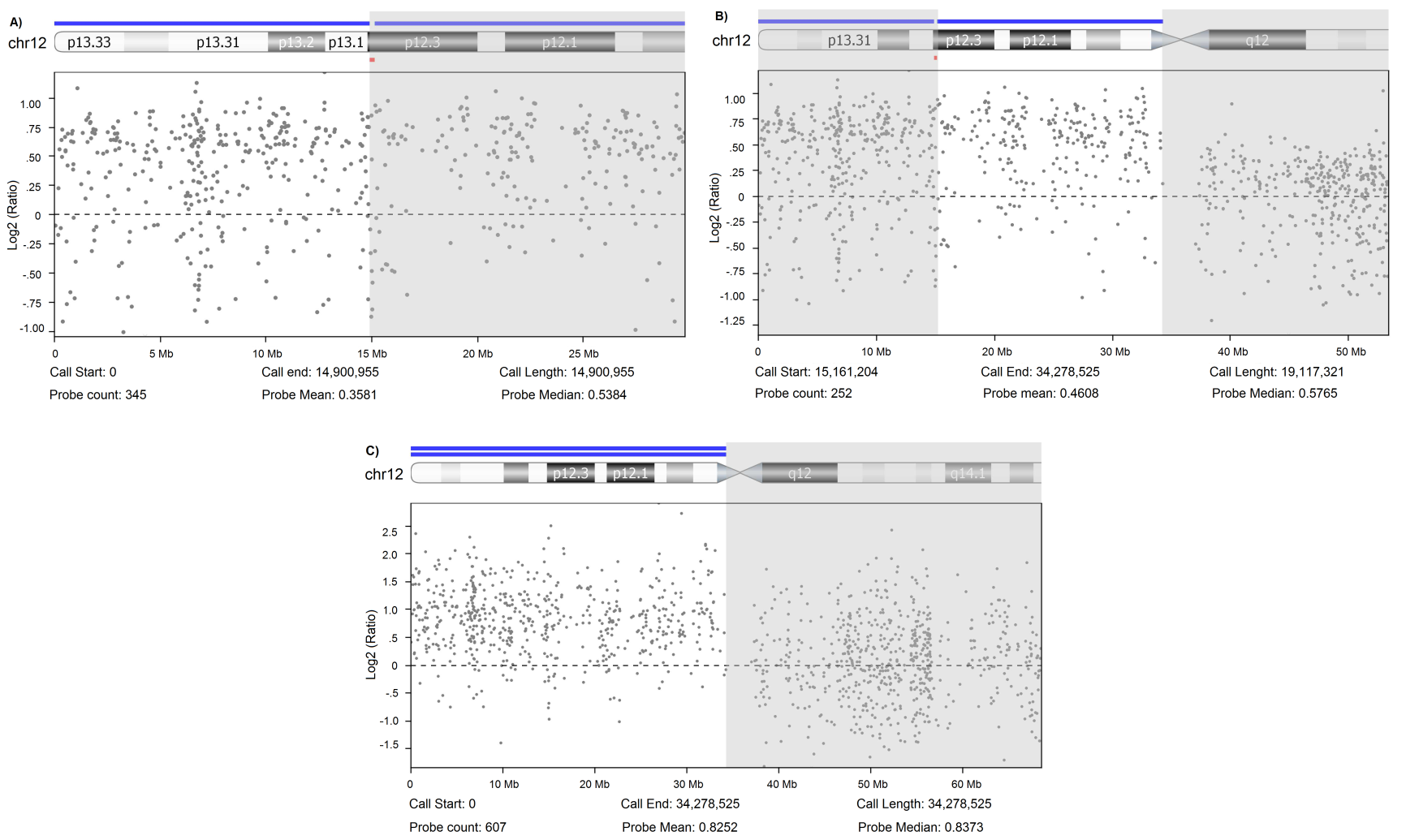
Supporting data**

**Figure S1. Hybridization profile for 12p amplification identified in tumor samples of twins.** In embryonal carcinoma (A-B) and the seminoma (C), gains at 12p were important novel CNV mapped at 12p amplicon. The 12p12.3-p11.1(A) and 12p13.33-p12.3 (B) were detected in twin-1 and 12p13.33-p11.1 (C) was the amplicon detect in Twin-2. Scatter plots with x-axis coordinate representing the probes positions along the genome. The top bars (blue) indicate gains of genomic regions, whereas the lower bars (red) indicate losses of genomic regions. The images were adapted from the Nexus 7.5 software program.
